# Associations between alexithymia, parental rearing styles, and frequency of drug use in male methamphetamine dependence patients

**DOI:** 10.1186/s12888-022-03897-0

**Published:** 2022-04-19

**Authors:** Cui Huang, Qiuyu Yuan, Shengya Shi, Menglin Ge, Xuanlian Sheng, Meng Yang, Ling Zhang, Lei Wang, Kai Zhang, Xiaoqin Zhou

**Affiliations:** 1 School of Mental Health and Psychological Sciences, Anhui Medical University, 69 Meishan Road, Hefei, 230032 Anhui Province China; 2 Department of Psychiatry, Chaohu Hospital of Anhui Medical University, 64 North Chaohu Road, Chaohu City, Hefei, 238000 China; 3 Anhui Psychiatric Center, 64 North Chaohu Road, Anhui Medical University, Hefei, 238000 China

**Keywords:** Methamphetamine, Frequency of drug use, Alexithymia, Parental rearing styles

## Abstract

**Background:**

Alexithymia, which is characterized by difficulty identifying and describing feelings, is a stable personality trait and it has been associated with early life experiences. Methamphetamine dependence patients with high level of alexithymia may be particularly vulnerable to engaging in more frequent methamphetamine use. Therefore, we aimed to evaluate whether alexithymia was associated with frequency of methamphetamine use. Additionally, the current study sought to examine early-life factors associated with the development of alexithymia, i.e., parental rearing styles.

**Method:**

Participants were 108 non-injecting methamphetamine dependent patients from a male compulsory detoxification center. The level of alexithymia was assessed by Toronto Alexithymia Scale-20(TAS-20). In addition, we applied Egna Minneu av. Bardndosnauppforstran (EMBU) to assess the parental rearing styles, including the dimensions of warmth, rejection, punishment/strictness, overinvolvement, overprotection, and favoring.

**Results:**

The total score of TAS-20 was positively correlated with frequency of methamphetamine use (*r* = 0.26, *p* < 0.01). Specifically, except for externally oriented thinking, difficulty identifying feelings (*r* = 0.23, *p* < 0.05) and difficulty describing feelings (*r* = 0.25, *p* < 0.05) were positively correlated with frequency of methamphetamine use. Multiple linear regression showed that more maternal rejection (B = 0.59, *p* = 0.002), or less maternal warmth (B = -0.22, *p* = 0.004) was associated with higher levels of alexithymia. Ordinal logistic regression showed that for every 1 score increase in the total score of TAS-20, there was a 1.06 times risk of a one level increase in the level of methamphetamine use frequency (OR = 1.06, *p* = 0.01).

**Conclusions:**

These results have major implications for understanding the role of alexithymia in craving and addiction, while providing a further and explicit entry point for addiction treatment. Moreover, more attention should be focused on parenting in relation to early experiences.

**Supplementary Information:**

The online version contains supplementary material available at 10.1186/s12888-022-03897-0.

## Introduction

Substance-related disorder is a prominent public health problem globally, which has complex pathophysiological and pathopsychological mechanisms [1]. Methamphetamine, as a highly addictive central nervous system stimulant, is associated with a range of health hazards, including mental disorders, somatic disorders, infectious diseases, and overuse [2, 3]. The prevalence and mortality rates associated with methamphetamine use have doubled in the past decade. Evidence suggested that methamphetamine use disorder could be the next drug use crisis after the opioid crisis worldwide [4].

Although multiple mechanisms exist for substance-related disorder, emotion-regulation dysfunctions are important factors in the onset, maintenance, recovery, and relapse of drug use [5]. Emotion processing, which includes emotion recognition, emotion regulation, and response, is closely related to the construct of alexithymia [6]. It is currently endorsed that alexithymia is the result of early emotion processing deficit rather than higher-order emotion regulation processes [7]. Alexithymia reflects inflexible emotion regulation, with underlying dysfunctions could occur in all phases of emotion processing, both psychologically and physically [8]. To be precise, alexithymia is characterized by difficulty identifying and describing feelings (one’s own and those of others), and an externally oriented cognitive style [9]. Considering that people with alexithymia have difficulty distinguishing various internal feelings from physical sensations, they complain about their various physical ailments, yet they are actually suffering from emotional distress [10]. Craving for methamphetamine is expressed as a physical “complaint” that is well identified by people with alexithymia.

Individuals with alexithymia appear to have difficulty coping with social and psychological stressors and are more inclined to use “addictive” patterns as a coping strategy to manage underlying feelings of distress [11]. Alexithymia has been recognized as an significant risk factor for a variety of psychological and behavioral problems, including maladaptive schemas [12], eating disorders [13], academic achievement [14], non-suicidal self-injury [15], etc. Furthermore, alexithymia has been widely associated with increased marijuana use [16], increased caffeine consumption [17], mobile phone addiction [11], and pathological gambling [18]. Psychiatrists began to focus on the characteristics of drug use, and a recent follow-up study found a link between alexithymia and relapses in patients with substance use disorders [19]. Previous research indicated that alexithymia was associated with more severe addictions such as increased substance use, intrusive and compulsive thoughts about substance use, and higher levels of cravings [20, 21]. Meanwhile, more severe alexithymia had been proved to be associated with more intense cue-elicited methamphetamine craving [22].

There is a controversy over whether alexithymia should be viewed as a stable personality trait or as a state-dependent phenomenon. Based on the accumulating research evidence on alexithymia, currently it is largely considered to be a personality trait of the general population throughout life [23]. Parents are the most important parts of one’s early life, and inappropriate parental rearing practices have lasting and detrimental impact on one’s mental and physical health [24]. Recent research suggested that parenting practices could influence adolescent adjustment [25]. Meanwhile, positive parental rearing styles can increase well-being by diminishing some of the potentially negative psychological traits of early adulthood [26]. Previous studies have shown significant differences in parental rearing styles among multiple substance dependent individuals compared to healthy individuals, primarily for rejection, lack of emotional warmth, and overprotection [27]. The perceived parental rearing styles of Internet-addicted adolescents were found to exhibited more overly intrusive, punitive, and unresponsive [28]. Besides, alexithymia was examined to display a mediating role between attachment and addictive behaviours [29]. Another study attempted to predict alexithymia from the perspective of attachment-related features, but this study was conducted with adult attention-deficit/hyperactivity disorder patients as the subjects and had significant flaws [30]. To our knowledge, only one documented study has linked inappropriate parental rearing styles to the initiation of drug use [31], yet there is a gap in research on the frequency of drug use.

Alexithymia is thought to be culturally relevant, and Asian cultures are perceived to be more inexpressive [32]. The parental rearing styles and emotional processing vary dramatically across cultures, and studies performed in Asian populations would be meaningful [33, 34]. Drug users most often initiate with non-injecting drug use and use drugs more and more frequently, then move on to injecting drug, which can have more serious consequences, such as infectious diseases and death [35, 36]. Therefore, studies addressing the frequency of drug use deserve more attention from clinicians and policy makers.

In view of the above, the objective of this study was to examine the relationship between alexithymia and frequency of drug use in methamphetamine dependence patients. As an exploratory analysis, we would investigate early life factors that shape alexithymia from the perspective of parental rearing styles.

## Methods

### Participants

All the participants were recruited from a male compulsory detoxification center in Anhui Province, China (from September to November 2019). Participants who met the following criteria were included in this study: (1) Han nationality, aged from 18 to 65 years; (; (2) met the Diagnostic and Statistical Manual of Mental Disorders, 5th edition (DSM-5) criteria for methamphetamine dependence; (3) were non-injecting drug users, and (4) were available to provide informed consent. Also, participants who met any of the following exclusion criteria were excluded: (1) suffering from severe psychiatric disorder, such as schizophrenia or bipolar disorder; (2) having used psychoactive drugs within the last month; and (3) those who have comorbidity with serious somatopathy (e.g., cardiovascular disease, neurological disease, diabetes, etc.)

### Measures

#### Sociodemographic and clinical variables

A self-designed questionnaire was used to collect and systematize the following variables: age, education level, marital status, income level, place of household registration, comorbidity, and addiction-related variables. The frequency of methamphetamine use was divided into 5 levels (level 1: about once per month or less; level 2: about once per 2 weeks; level 3: about once per week; level 4: about once per 1 to 3 days; level 5: about several times per day).

### Alexithymia

The Toronto Alexithymia Scale-20 (TAS-20) is a self-report scale that was designed to assess alexithymia level [37]. The total score of TAS-20 ranged from 20 to 100, with higher scores representing a higher level of alexithymia. TAS-20 consists of three dimensions, including difficulty identifying feelings, difficulty describing feelings, and externally oriented thinking. TAS-20 has proved to be a reliable and effective tool for assessing alexithymia in the Chinese population [38, 39]. The recommended cut-off score for the Chinese population is a total score of 57 or above indicating a high level of alexithymia [39, 40]. The Cronbach’s alpha was reported to be 0.81.

### Parental rearing styles

The original version of the Egna Minneu av. Bardndosnauppforstran was designed to assess participants’ perceived parental rearing styles in childhood [41], which was translated into Chinese and showed good reliability and validity [42, 43]. EMBU contains 66 items, including 11 factors, including M-warmth, F-warmth, M-rejection, F-rejection, M-Punishment/strictness, F-Punishment/strictness, M-overinvolvement-overprotection, F-overinvolvement, F-overprotection, M-favoring, F-favoring (M for mother, F for father). The Cronbach’s alpha was reported to be 0.92.

### Procedures

Prior to the start of the research process, detailed information about the study was explained to the participants and we ensured that the participants could fully understood the study. Participants did not receive any financial compensation. Each participant had to sign an informed consent form and each participant could withdraw from this study at any stage of the study if they wished. During the study, we used questionnaires and structured talks, and no invasive or harmful tests were used, nor were any drugs applied. The Ethics Committee of Chaohu Hospital of Anhui Medical University approved the study (Ethics number: 201901- kyxm-02). All study procedures were in accordance with the Helsinki Declaration.

### Statistical analysis

The IBM SPSS21.0. was applied for statistical analysis. Firstly, the Kolmogorov-Smirnov single-sample test was used to examine the type of data distribution. Next, independent sample t test or Mann Whitney U test was used to compare the continuous variables between H-alexithymia (high levels of alexithymia, TAS-20 ≥ 57) group and L-alexithymia (low levels of alexithymia, TAS-20 < 57) group. The Chi-square test was performed for categorical variables. Pearson correlations or Spearman correlations analysis was used to explore the correlations between alexithymia, parental rearing styles, and frequency of methamphetamine use. In addition, multiple linear regressions were used to analyze the effects of parental rearing styles on alexithymia. As a final step, ordinal logistic regressions were used to analyze the risk of alexithymia on the frequency of methamphetamine use. Statistical significance was defined as *p* < 0.05, and all reported *p* values were bilateral.

## Result

### Characteristics of methamphetamine dependence patients

There were 123 participants eligible to participate in the study, and after screening, 108 methamphetamine dependent individuals were eventually included in this study. Of the 15 excluded participants, 8 were heroin users, and 7 submitted incomplete data.

The characteristics of methamphetamine dependent patients are shown in Table 1. The current age of the total participants ranged from 22 to 47 years (30.8 ± 3.9 years), and the mean age of H-alexithymia group (29.3 ± 3.3) was younger than that of L-alexithymia group (31.8 ± 4.0). Moreover, the age of first drug use of H-alexithymia group (21.2 ± 3.9) was earlier than L-alexithymia group (23.5 ± 4.4) (t = − 2.78, *p* < 0.01). The frequency of drug use was found to be significantly more frequent in H-alexithymia group than in L-alexithymia group (Z = 2.15, *p* = 0.03). In addition, no differences were found between the two groups in terms of education level, place of household registration, income level, and marital status.

**Table 1:** Characteristics of methamphetamine dependence patients (*n* = 108)

Variables	H-alexithymia(*n* = 44)	L-alexithymia(*n* = 64)	t/Z/λ	*p*
Age(years)	29.3(3.3)	31.8(4.0)	−3.41	<0.01
Age of first drug use(years)	21.2(3.9)	23.5(4.4)	−2.78	<0.01
Education level(years)	8.0(2.7)	8.5(3.0)	−0.92	0.36
Place of household registration			0.01	0.92
Urban areas	25(40.3%)	37(59.7%)		
Rural areas	19(41.3%)	27(58.7%)		
Income level(CNY)			0.14	0.71
≥ 3000	33	50		
< 3000	11	14		
Marital status			2.67	0.26
Unmarried	22	22		
Married	12	24		
Divorced	10	18		
Frequency of drug use(monthly)*	17.07(12.00)	12.08(11.65)	2.15	0.03

Note: *p < 0.05, **p < 0.01; H-alexithymia, high level of alexithymia; L-alexithymia, low level of alexithymia. Values are presented as number (%) or mean (standard deviation)

*Here ‘frequency of drug use’ was transformed into times(monthly): level 1, as 1 time per month; level 2, as 2 times per month; level 3, as 4 times per month; level 4, as 15 times per month; level 5, as 30 times per month

### Correlations between alexithymia, parental rearing styles and frequency of methamphetamine use

Table 2. presented the results of the correlation analyses between all the variables. The total score of alexithymia was positively correlated with the frequency of methamphetamine use (*r* = 0.26, *p* < 0.01). Specifically, difficulty identifying feelings (*r* = 0.23, *p* < 0.05) and difficulty describing feelings (*r* = 0.25, *p* < 0.05) were positively correlated with the frequency of methamphetamine use, while externally oriented thinking was not found to be statistically correlated with the frequency of methamphetamine use. In addition, parental rearing styles of rejection (M-: *r* = 0.42, *p* < 0.01; F-: *r* = 0.28, < 0.01) and Punishment/strictness (M-: *r* = 0.33, *p* < 0.01; F-: *r* = 0.33, *p* < 0.05) were positively associated with alexithymia. In contrast, maternal rearing styles of warmth (*r* = 0.30, *p* < 0.01) was negatively associated with alexithymia.

**Table 2:** Correlations between alexithymia, parental rearing styles and frequency of methamphetamine use

Variables		M(SD)	Alexithymia	Difficulty Identifying Feelings	Difficulty Describing Feelings	Externally Oriented Thinking
Frequency of methamphetamine use(monthly)			0.26^**^	0.23^*^	0.25^*^	0.08
M-	Warmth	53.26(9.52)	−0.30^**^	−0.24^*^	−0.16	−0.25^**^
F-	Warmth	47.94(9.82)	−0.18	−0.18	− 0.041	− 0.20^*^
M-	Rejection	12.36(3.94)	0.42^**^	0.36^**^	0.24^*^	0.26^**^
F-	Rejection	9.63(3.08)	0.28^**^	0.32^**^	0.18	0.07
M-	Punishment/strictness	11.61(3.46)	0.33^**^	0.29^**^	0.22^*^	0.21^*^
F-	Punishment/strictness	17.95(5.23)	0.25^*^	0.27^**^	0.19	0.10
M-	Overinvolvement-	37.78(6.95)	0.18	0.22^*^	0.02	0.08
	Overprotection					
F-	Overinvolvement	21.28(3.84)	0.06	0.09	0.06	−0.028
F-	Overprotection	10.71(2.64)	0.10	0.11	0.09	− 0.028
M-	Favoring	13.26(4.03)	−0.07	−0.03	− 0.01	−0.10
F-	Favoring	12.38(4.20)	−0.02	0.01	−0.03	−0.05

Notes: *p < 0.05, **p < 0.01; *M(SD)* mean (standard deviation), *M-* mother, *F-* father

### Multiple linear regressions of parental rearing styles and alexithymia

Multiple linear regression was used to analyze the effect of parental rearing styles on alexithymia. Using a stepwise approach, M-warmth and M-rejection were entered into the final regression model(*R*^2^ = 0.18). Table 3 showed that more rejection (B = 0.59, *p* = 0.002), or less warmth (B = -0.22, *p* = 0.004) in the mother’s parenting practices was associated with higher levels of alexithymia.

**Table 3:** Multiple linear regressions of parental rearing styles and alexithymia

		B	SD	t	*p*
M-	Rejection	0.59	0.18	3.23	0.002
M-	Warmth	−0.22	0.08	−2.98	0.004

### Ordinal logistic regressions of alexithymia and frequency of methamphetamine use

Ordinal logistic regression was applied to analyze the risk of alexithymia on the frequency of methamphetamine use. Table 4 showed that for every 1 score increase in the total score of alexithymia, there was a 1.06 times risk of a one level increase in the level of methamphetamine use frequency (OR = 1.06, *p* = 0.01).

**Table 4:** Odds ratios for the association of alexithymia with frequency of methamphetamine use by ordinal logistic regression

	B	SE	Wald	OR	95%Exp(B)		*p*
					Lower	Upper	
Alexithymia	0.06	0.02	6.42	1.06	1.01	1.11	0.01

## Discussion

This study examined whether severe alexithymia was associated with more frequent drug use and the contributions of specific parental rearing styles to the formation of alexithymia among male methamphetamine dependence patients. The results showed that methamphetamine users with high levels of alexithymia used drugs more frequently, and more precisely, alexithymia was positively associated with the frequency of drug use. Furthermore, we explored possible parental rearing factors relevant to the formation of alexithymia. Our findings indicated that more maternal rejection and less maternal warmth contributed to high levels of alexithymia.

The relationship between alexithymia and addictions has been documented and discussed many times, including in such fields as heroin dependent [44], alcohol addiction [20], internet addiction [9], pathological gambling [45], etc.

However, to the best of our knowledge, very few studies have focused on specific characteristics of addiction, i.e., frequency of substance use. Our current study showed that alexithymia was significantly associated with the frequency of methamphetamine use. Based on online questionnaire surveys among people with pain in the general population, alexithymia is associated with frequently painkiller use, which supported our findings [46]. Alexithymia mainly characterized by difficulty in identifying feelings, which can be perceived as a deficit in cognitive processing and emotional regulation [47].

The frequency of drug use reflects a degree of craving, and patients with high subjective cravings will repeatedly self-administer drugs. One study on alcohol consumption found that alexithymia is associated with subjective hypersensitivity to bodily sensations, and difficulty identifying feelings mediated the relationship between sensitivity to bodily sensations and alcohol consumption [44]. Consistent with our research, one study tested cue-elicited craving responses in methamphetamine-dependent individuals and found a positive correlation between the difficulty identifying feelings and cue-elicited craving responses [22]. Although there have been no conclusive researches between drug cravings and alexithymia, there have found that alcohol craving was associated with alexithymia in alcohol-dependent patients [48]. Meanwhile high level of alexithymia was associated with significantly higher rates of higher levels of desire for alcohol and alcohol consumption [49]. Although there are similarities in the brain regions involved in each stage of the addiction cycle, there are differences in the specific mechanisms and psychopathology concerned by different substance addictions [50]. Our study effectively addressed the previous gap, that is, the association of alexithymia with frequent drug use. Besides, our data did not support the correlation between externally oriented thinking and the frequency of methamphetamine use, but it seemed to be well understood. Despite TAS-20 has been worldwide used in researches and clinical practices, a critical review of TAS-20 revealed that, in practically all studies, the dimension ‘externally oriented thinking (‘appears to be unreliable [51].

Regarding the relationship between alexithymia and parental rearing styles of methamphetamine dependence patients, we found that the development of alexithymia was associated with malpractice in parenting, mainly in the form of rejection, punishment/strictness, and warmth. In fact, warmth and rejection belong to opposite poles on one dimension. Punishment/strictness refers to strict parents who would punish their children roughly or in embarrassing ways for very small things or for no reason at all, and these parents tend to punish their children more than they deserve. In the Spanish population, parenting styles characterized by less warmth were associated with poorer psychosocial adjustment. However, indulgent parenting style (warmth but not strictness) was associated with equal or even better outcomes in terms of psychosocial adjustment than authoritative parenting (warmth and strictness) [52, 53]. Yet studies of adolescents from middle-class European-American families had shown that authoritative parenting (warmth and strictness) is most beneficial to adolescent development, but with a higher degree of internalized distress that manifests as complaints of somatic discomfort [54] Under strict parenting styles, children suppress and internalize their distressed feelings rather than choosing to express them, yet internally focused feelings and complaints about somatic discomfort represent high levels of alexithymia. The emerging view is that warmth but not strictness parental rearing styles appears to be the optimal parental rearing style, which can facilitate the psychosocial development in children [55]. As far as we know, the present study is the first to report the relationship between parental rearing styles and alexithymia among drug users. There are many possible factors that can explain the links between parental rearing styles and alexithymia. Improper parenting has been linked to the development of childhood trauma, and childhood trauma was a direct predictor of alexithymia [36, 56]. Furthermore, childhood emotional abuse was proved to be a risk factor for alexithymia in male substance dependence inpatients [57]. From another perspective, the frequent rejection and punishment of children by parents can be considered as a form of abuse. And on that basis, perceived parental rejection has been linked to insecure attachment, meanwhile, attachment styles would interact with alexithymia and elevate the risk of substance-related disorder [58, 59]. Additionally, parental rearing patterns of Chinese individuals with borderline personality exhibited greater rejection, punishment, and less emotional warmth, and that maternal emotional warmth may be an important protective factor against the development of borderline personality [60]. Moreover, individuals with borderline personality manifest higher levels of alexithymia [61], and they showed diminished accuracy in recognition of emotions in computerized tasks [62]. Given that substance-related disorders and borderline personality are common clinical comorbidities, Gratz et al. examined the complex relationship between substance-related disorders, borderline personality, childhood abuse, and emotion dysregulation among substance users, and found that borderline personality was associated with higher levels of childhood maltreatment, and that emotion dysregulation played a mediating role between childhood maltreatment and borderline personality [63]. Also, using path model analysis, Tom demonstrated that anxious attachment partially mediated the relationship between alexithymia and craving [64].

The extrinsic association between alexithymia and the frequency of drug use has a substantial intrinsic biological basis, and although the direct relationship has not been confirmed, the indirect relationship could be well understood. Brain structures involved in emotion processing have been investigated for functional and structural alterations associated with alexithymia. The fusiform gyrus and anterior cingulate cortex have been demonstrated repeatedly to be areas with functional and structural abnormalities associated with alexithymia [65, 66]. Recent researches suggested that the pathway by which childhood adversity leads to substance use in late adolescence may operate through hypofunctioning of the anterior cingulate cortex associated with inhibitory control and externalizing behavior [67]. The neurotransmission through dopamine D2-type receptors in the anterior cingulate and anterior insular cortex affects emotional processing in healthy individuals, yet this association was observed to be absent in methamphetamine dependence patients by positron emission tomography [68]. The fusiform gyrus is responsible for processing of faces and therefore for emotion recognition [69]. Voxel-based morphometry revealed that the density around the left fusiform gyrus of cannabis users differed from that of non-users [70].

Alexithymia was associated with a dampened basal activity of the thalamus-pituitary-adrenal (HPA) axis, the main endogenous stress response system [71]. Emotion-related physiological arousal-cortisol reactivity-and subjective emotion regulation have been independently linked to substance use. Parental acceptance-rejection is a primary factor in the establishment and development of attachment relationships [59], and individuals with insecure attachment show a reduced cortisol awakening response [72]. A study based on a stressful parent-child interaction task showed that adolescents with low levels of cortisol reactivity and high difficulties with emotion regulation were more likely to use substances [73].

On a more rigorous level, our study has some limitations. Firstly, this was a cross-sectional survey and we could not infer that alexithymia caused drug users to take drugs more frequently. Perhaps, in a dramatic reversal, drug users exhibit high levels of alexithymia, because frequently drug use could impair emotional functioning. Hence, higher levels of alexithymia could be a precursor of frequent drug use, or a result of frequent drug use, or both. Rigorous follow-up studies are needed to statistically clarify this association. Secondly, assessing perceived parental rearing styles was a method of retrospective recall, which may lead to recall bias. Thirdly, our sample size was limited, so a large sample size will be more convincing. Additionally, our current study did not include a control group, and a controlled study would provide more clarity and insight into the important role of alexithymia. Finally, the participants in this study were all Han Chinese adult males, and extreme caution should be taken when generalizing to other cultural contexts for parenting pattern recommendations.

## Conclusion

Despite these limitations, the current study confirmed the association between alexithymia and the frequency of drug use, as well as revealed that parental rearing styles had an important contribution to the formation of alexithymia. Our findings have major implications for understanding the role of alexithymia in craving and addiction, while providing a further and explicit entry point for addiction treatment. Our results supported the debate about treatment approaches targeting the transdiagnostic process of alexithymia in substance dependence. Moreover, more attention should be focused on parenting in relation to early experiences. Applying family-related factors (i.e., parental rearing styles) of alexithymia could facilitate a tailored approach in psychiatry and psychotherapy.

## Supplementary Information

**Additional file 1.**

## Data Availability

The datasets generated and analyzed during the current study are not publicly available due to no permission from participants to share anonymized participant data publicly but are available from the corresponding author on reasonable request.

## Abbreviations

TAS-20: Toronto Alexithymia Scale-20
EMBU: Egna Minneu av. Bardndosnauppforstran
M-: Mother
F-: Father
