# Supplementary material for: Associations between alexithymia, parental rearing styles, and frequency of drug use in male methamphetamine dependence patients
Source: BMC Psychiatry. 2022 Apr 19;22:276. doi: 10.1186/s12888-022-03897-0 (PMC9020116; doi:10.1186/s12888-022-03897-0)
Supplement: Supplementary file 1 — Additional file 1. [file 12888_2022_3897_MOESM1_ESM.docx]

Correlations between frequency of methamphetamine use and parental rearing styles.

| Frequency of methamphetamine use | | r | *p* |
| --- | --- | --- | --- |
| M- | Warmth | 0.08 | 0.40 |
| F- | Warmth | 0.03 | 0.76 |
| M- | Rejection | 0.12 | 0.24 |
| F- | Rejection | -0.01 | 0.95 |
| M- | Punishment/strictness | 0.11 | 0.28 |
| F- | Punishment/strictness | -0.02 | 0.86 |
| M- | Overinvolvement- | 0.24 | 0.01 |
|  | Overprotection |  |  |
| F- | Overinvolvement | -0.05 | 0.64 |
| F- | Overprotection | -0.02 | 0.87 |
| M- | Favoring | 0.09 | 0.40 |
| F- | Favoring | 0.03 | 0.73 |
